# Study of the Dilution-Induced Deposition of Concentrated Mixtures of Polyelectrolytes and Surfactants

**DOI:** 10.3390/polym14071335

**Published:** 2022-03-25

**Authors:** Laura Fernández-Peña, Eduardo Guzmán, Coral Fernández-Pérez, Irene Barba-Nieto, Francisco Ortega, Fabien Leonforte, Ramón G. Rubio, Gustavo S. Luengo

**Affiliations:** 1 Departamento de Química Física, Facultad de Ciencias Químicas, Universidad Complutense de Madrid, Ciudad Universitaria s/n, 28040 Madrid, Spain; laura.fernandez.pena@ucm.es (L.F.-P.); cferna01@ucm.es (C.F.-P.); irebarba@ucm.es (I.B.-N.); fortega@quim.ucm.es (F.O.); 2 Centro de Espectroscopía y Correlación, Universidad Complutense de Madrid, Ciudad Universitaria s/n, 28040 Madrid, Spain; 3 Instituto Pluridisciplinar, Universidad Complutense de Madrid, Paseo Juan XXIII 1, 28040 Madrid, Spain; 4 L’Oréal Research and Innovation, 1 Avenue Eugène Schueller, 93600 Aulnay-Sous-Bois, France; fabien.leonforte@rd.loreal.com

**Keywords:** polyelectrolyte, surfactant, solid surface, deposition, precipitation, dilution

## Abstract

Mixtures of polyelectrolytes and surfactants are commonly used in many technological applications where the challenge is to provide well-defined modifications of the surface properties, as is the case of washing formulations in cosmetics. However, if contemporary experimental and theoretical methods can provide insights on their behavior in concentrated formulations, less is known on their behavior under practical use conditions, e.g., under dilution and vectorization of deposits. This makes it difficult to make predictions for specific performance, as, for example, good hair manageability after a shampoo or a comfortable sensorial appreciation after a skin cleanser. This is especially important when considering the formulation of new, more eco-friendly formulations. In this work, a detailed study of the phase separation process induced by dilution is described, as well as the impact on the deposition of conditioning material on negatively charged surfaces. In order to gain a more detailed physical insight, several polyelectrolyte–surfactant pairs, formed by two different polymers and five surfactants that, although non-natural or eco-friendly, can be considered as models of classical formulations, have been studied. The results evidenced that upon dilution the behavior, and hence its deposition onto the surface, cannot be predicted in terms of the behavior of simpler pseudo-binary (mixtures of a polymer and a surfactant) or pseudo-ternary mixtures (two polymers and a surfactant). In many cases, phase separation was observed for concentrations similar to those corresponding to the components in some technological formulations, whereas the latter appeared as monophasic systems. Therefore, it may be assumed that the behavior in multicomponent formulations is the result of a complex interplay of synergistic interactions between the different components that will require revisiting when new, more eco-sustainable ingredients are considered.

## 1. Introduction

Formulations in many technological applications, e.g., cosmetics (washing formulations, including different gels and shampoos), food science, pharmacy, tertiary oil recovery, are very complex mixtures which contain, among many other components, several surfactants bearing charges of different signs (commonly anionic, zwitterionic, and neutral), and polymers (mainly positively charged polyelectrolytes) [1]. The mutual interactions (commonly electrostatic, hydrophobic, or hydrogen bond) between polymer chains and surfactant molecules lead to the formation of soluble supramolecular complexes in solution [2,3,4], which results in the formation of one-phase systems (1ϕ). These single-phase concentrated mixtures can undergo phase-separation processes upon dilution, which can take place during practical use. It has been previously shown that two-phase systems (2ϕ) are also obtained when the surfactant concentration is increased from highly diluted surfactant + polyelectrolyte mixtures [5,6,7,8,9]. This phase separation leads to the depletion of colloidal aggregates (solid-like or liquid-like) from the aqueous phase, resulting in their deposition on the solid surface which in turn is fundamental for defining the final performance of the formulations [10,11,12,13]. Therefore, it is possible to assume that the performance of most of the currently commercialized polyelectrolyte + surfactant formulations is mediated by the appearance of an enhanced deposition as the result of a dilution-induced precipitation process, the so-called Lochhead effect [1,14,15]. This situation is very different to the enhanced deposition occurring when aqueous mixtures of oppositely charged polyelectrolytes and surfactant undergo a phase separation process as the result of the increase of the surfactant concentration up to a concentration equivalent to that of the charged monomers [16,17,18]. Whereas for the latter case, the enhanced deposition occurs by the depletion of neutral polyelectrolyte–surfactant complexes as a result of their lacking colloidal stability [19], the deposition enhanced by dilution is a more complex situation, which occurs by changing the total solution concentration without any change on the ratio between the number of surfactant molecules and charged monomers in the solution. Therefore, the enhancement of the deposition is due to a complex interplay between the different interactions operating within the polyelectrolyte–surfactant mixture [20,21]. This is especially complex for multicomponent systems, as those involved in technological applications, e.g., shampoos, emulsions, foams, for which the composition corresponding to the onset on the phase separation is difficult to predict [22,23].

Control of the phase separation induced by dilution is a challenge for most formulators because a poor compatibility between the different components of the mixture may reduce the efficiency of the formulation [24]. Therefore, it is necessary to deepen on the understanding about the intricate balance of interactions that emerge between polymers and surfactants in any formulation to find suitable solutions for the design of optimal products [25,26]. This work attempted to provide a physico-chemical perspective to the performance of concentrated polyelectrolyte–surfactant mixtures by studying their phase behavior and their adsorption onto negatively charged solid surfaces that have physico-chemical characteristics analogous to those expected during hair shampooing or an oil recovery process from rocky reservoirs [15,27]. It should be noted that the systems studied here are models that do not fulfill the current eco-sustainability demands of the industry. However, the understanding of the performance of these models plays an essential role in seeking suitable combinations of bio-based and eco-friendly polymers and surfactants for the design of formulations for specific applications, providing valuable information in the choice of new eco-sustainable ingredients [28].

## 2. Materials and Methods

### 2.1. Chemicals

Polydiallyl-dimethyl-ammonium chloride, (PDADMAC) with a molecular weight in the 100–200 kDa range, and the zwitterionic copolymer Merquat™ 2003 (copolymer of acrylic acid, 3-Trimethylammonium propyl methacrylamide chloride and acrylamide in the molar ratio 10:40:50) with a molecular weight of about 1200 kDa were purchased from Sigma-Aldrich (Saint Louis, MO, USA) and Lubrizol (Wickliffe, OH, USA), respectively. Polymers were used as received without any further purification.

**Figure 1:**
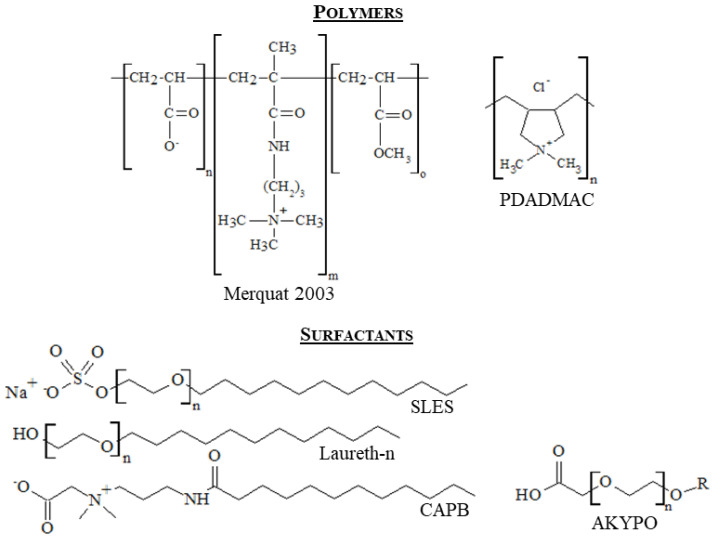
Molecular structures of the different polymers and surfactants used in this study. n assumes different values depending on the surfactant. n = 2 for SLES, n = 4 for AKYPO, n = 4 or 12 in Laureth-n for Laureth-4 and Laureth-12. On the other side, R corresponds to alkyl chains containing 12 carbons (dodecyl) in AKYPO.

Five different surfactants were explored to obtain concentrated polyelectrolyte-surfactant formulation with possible technological application. Laureth-5 carboxylic acid (AKYPO) and sodium lauryl ether sulfate (SLES) was supplied by Kao Chemicals Europe, Barcelona, Spain). Cocamidopropyl betaine (CAPB) purchased from Solvay (Brussels, Belgium). Two different lauryl ethers with a different number of oxyethylene groups (4 and 12), Laureth-4 (LE4) and Laureth-12 (LE12), were supplied by Ecogreen Oleochemicals (Dessau-Roßlau, Germany). Figure 1 shows the molecular formula of the different polymers and surfactants used in this study.

Ultrapure deionized water used for cleaning and solution preparation was obtained by a multicartridge purification system aquaMAX^TM^-Ultra 370 Series (Young Lin Instrument, Co., Anyang, Korea). The water used had a resistivity higher than 18 MΩ∙cm, and a total organic content lower than 6 ppm. Citric acid (purity 99.9%) and NaCl (purity 99.95%) purchased from Sigma-Aldrich (Saint Louis, MO, USA) were used for fixing the pH and the ionic strength of the polyelectrolyte–surfactant mixtures.

### 2.2. Model Technical Formulation

An experimental multicomponent formulation of a commercial shampoo is shown in Table 1 [29]. Typical concentrations of the polymers are 0.2 to 0.5% *w*/*w*, whereas those of the surfactants are between 20 and 250 mM.

Typical formulations used for enhanced oil recovery have been recently reported by Ramos et al. [30]. These present concentrations of polymers and surfactants range between 0.1 and 0.3% *w*/*w* and 100 and 250 mM, respectively, and in general brine is used instead of pure water, which allows fixing the ionic strength. Considering the above aspects, a model multicomponent mixture was designed using a combination of polymers and surfactants studied in our previous works [31,32,33,34]. Furthermore, different pseudo-binary (polyelectrolyte–surfactant mixtures) and pseudo-ternary mixtures (two polymers and a surfactant), maintaining similar composition to those abovementioned and to that of the designed multicomponent mixture, were also studied. Table 2 reports the composition of the designed multicomponent model mixture.

The preparation of the different experimental polymer–surfactant mixtures was performed following a crude mixing protocol adapted from our previous publications [5,25,32]. This methodology can be summarized as follows: (i) the required amounts of aqueous stock solutions of the polymers were weighed and poured into a flask to prepare the final mixture containing a total PDADMAC and/or Merquat 2003 concentrations of 0.5% *w*/*w* and 0.25% *w*/*w*, respectively. Afterwards, NaCl (purity 99.95%) was added to reach a salt concentration in the final mixture of 120 mM. The last step was the addition of the surfactant solution (pH~4.5) and a subsequent dilution with a citric acid solution of pH~4.5 up to reaching the desired surfactant concentration. It is worth mentioning that for the polyelectrolyte–surfactant solution preparation, the surfactant was added from solutions having a concentration one order of magnitude higher than that of the final mixture. The different components were successively and quickly added, and the solutions were homogenized under mild stirring conditions (1000 rpm). It was expected that this procedure would lead to kinetically-trapped aggregates during the initial mixing, as it has been commonly described for polyelectrolyte–surfactant mixtures [35,36,37,38]. In order to take into consideration any trapped structures, and to ensure reproducibility, all samples were allowed to age during one week before their use at 25 °C. Considering that citric acid is not a buffer, the pH was measured after the aging period, and just before the utilization of the samples, to ensure that samples were always measured at pH = 4.5. All the experiments were carried out at 25.0 ± 0.1 °C.

It is worth mentioning that the values of pH and ionic strength were in the range of those used in most hair care cosmetic treatments used for reducing the bleaching of hair fibers [15,39,40]. Therefore, it is expected that the results obtained here can contribute to the understanding of the phenomena occurring during the application of shampoos under the shower.

### 2.3. Characterization of the Adsorption onto Solid Surfaces

A quartz-crystal microbalance with dissipation monitoring (QCM-D) from KSV (Model QCM Z-500, Espoo, Finland) fitted with gold-coated AT-cut quartz crystals (Note: gold-coated AT-cut quartz crystals were initially cleaned with piranha solution, 70% sulfuric acid/30% hydrogen peroxide, over thirty minutes, and then thoroughly rinsed with Milli-Q water) was used for evaluating the adsorption of polyelectrolyte–surfactant layers. The surface of the quartz sensor was modified by a self-assembled monolayer of 3-mercapto-propanesulfonic acid to obtain a negatively charged surface. QCM-D measures the impedance spectra of a quartz crystal for the fundamental frequency (*f*_0_ = 5 MHz) and the odd overtones up to the 11th (central frequency of the 11th overtone, *f*_11_ = 55 MHz). The obtained impedance spectra were analyzed using a single-layer model following the procedure described by Voinova et al. [24], which provides the effective acoustic thickness or hydrodynamic thickness of the adsorbed layer, *h*_ac_. This procedure makes it possible to correlate the changes in the resonant frequency Δ*f* and dissipation factor Δ*D* of the different overtones with the physical parameters of the layers (thickness *h*_j_, density *ρ*_j_, elasticity *μ*_j_ and viscosity *η*_j_). Further details on the data analysis can be found in our previous publication [31].

An imaging null-ellipsometer from Nanofilm (Model EP3, Göttingen, Germany) was also used to determine the amount of material adsorbed onto the solid surfaces as the optical thickness, *h*_op_. Ellipsometry experiments were carried out using a solid–liquid cell at a fixed angle of 60° using silica plates as substrate (Siltronix, Archamps, France). These substrates were treated with piranha solution for 30 min to create a charged surface similar to that of thiol-decorated gold surfaces [41]. The experimental variables measured in ellipsometry are the ellipsometric angles, Δ and *Ψ*, which are related to the ratio between the reflection coefficients for the parallel (*r*_p_) and normal (*r*_s_) components of the magnetic field derived by Fresnel, i.e., to the ellipticity *ρ*^e^ [42,43]. The optical thickness, *h*_op_, and refractive index of the adsorbed layers are obtained from the experimental measurements by assuming a slab model describing the system according to the procedure described in [44]. Once the slab is defined, the thickness and the refractive index are obtained as the pair of values that minimize the differences between the experimental values of the ellipsometric angles and those obtained solving the Fresnel’s equation using the four-layer model [45,46].

*h*_ac_ and *h*_op_ should not be considered as absolute thicknesses due to the heterogeneity of most of the polyelectrolyte layers; thus, any discussion contained in this work considered *h*_ac_ and *h*_op_ as effective thicknesses that provided different information about the adsorbed amount within the layer [45,47]. The combination of ellipsometry and QCM-D is important because of the different sensitivities of these techniques to the water. This is because whereas the QCM-D provides information on the total mass of the adsorbed layer, including both the polymer and the water associated with such a layer, ellipsometry, which is based in the differences between the refractive indices of the layer and the medium, only gives information on the amount of adsorbed polymer. This difference leads to *h*_op_ ≤ *h*_ac_ and allows one to estimate the water content of the layers *x*_w_ as [32,41]

(1) $$x_{w}=\frac{h_{\mathrm{ac}}-h_{\mathrm{op}}}{h_{\mathrm{ac}}}$$

AFM measurements of dry layers deposited onto SiO_2_ substrates were carried out in air at room temperature using a NT-MDT Ntegra Spectra (NT-MDT Spectrum Instruments Ltd., Moscow, Russia) in the tapping mode using a silicon tip, model RTESP (Veeco Instrument Inc., Painview, NY, USA). It is worth mentioning that even though some changes in the morphology could be expected due to the drying process, the general aspects obtained from the analysis of wet and dry samples should not significantly change the conclusions extracted from the images [6].

The model surfaces used in the present study were negatively charged, presenting a ξ-potential values around −42 ± 5 mV (obtained from the measurement of the ξ-potential of colloidal particles with the same surface nature as the flat model surfaces [44]). Such value is in good agreement with those found for the ξ-potential of damaged hair fibers, from −55 to −35 mV depending on their origin and chemical treatments [40]. Therefore, model surfaces can be used to mimic the hair fiber surface, at least from a physico-chemical perspective.

## 3. Results and Discussion

### 3.1. Determination of the Phase Separation upon Dilution for Concentrated Pseudo-Binary Polymer–Surfactant Mixtures

**Figure 2:**
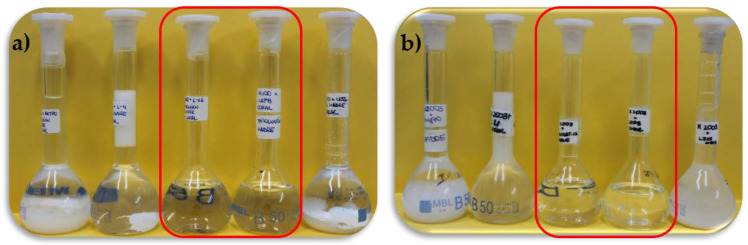
Set of images showing the aspect of the pseudo-binary polyelectrolyte–surfactant mixtures with the same composition of the components as in Table 2: (**a**) PDADMAC–surfactant mixtures; (**b**) Merquat 2003–surfactant mixtures. In both panels, the mixtures contain the following surfactants (from left to right): AKYPO, LE4, LE12, CAPB, and SLES. One-phase mixtures are surrounded by a red rectangle.

The first step of the study was focused on the determination of the pseudo-binary polyelectrolyte–surfactant concentration range for which the mixtures were in the one-phase region. This required us to study up to 10 independent polyelectrolyte–surfactant pairs, which corresponded to five polymer–surfactant pairs for each considered polymer. Figure 2 reports the aspect of the different pseudo-binary polymer–surfactant mixtures with the same composition of the components as that existing in the formulation shown in Table 2.

The combination of PDAMAC or Merquat 2003 with the five surfactants did not always lead to the formation of one-phase systems (1ϕ), and phase-separated systems (2ϕ) were obtained for most of the polyelectrolyte-surfactant pairs. This phase separation is evidenced by the appearance of a solid-like phase (precipitation) which settles at the bottom of the flask (mixtures containing AKYPO or SLES) or by the formation of two well-separated liquid phases with different density, i.e., coacervation, as occurs for the mixtures containing LE4. On the other side, mixtures of both polymers with CAPB and LE12 appear as 1ϕ. It should be noted that polymers and surfactants with the concentrations used for preparing the mixtures are perfectly soluble in water.

The origin of the phase separation in the polyelectrolyte–surfactant pairs was not straightforward, and deserved a specific analysis. For the case of the mixtures of the polymers with AKYPO and SLES, it was true that the surfactant molecules could bind electrostatically to the PDADMAC monomer and the positively charged monomers of Merquat 2003. This resulted in a significant charge reduction, and increased the hydrophobicity of the polyelectrolyte–surfactant complexes, which may destabilize the solution, thus leading to phase separation [34,48]. However, for the case of PDADMAC, the ratio between the concentrations of surfactant and charged monomers (S/P ratio) clearly exceeded the unity, i.e., the neutralization point, assuming a value of about 5, which should lead to the formation of overcharged negative complexes. Similarly for the binding of AKYPO to PDADMAC, a S/P ratio of about 3 was expected which should also take the system to a one-phase region of the phase diagram characterized by the presence of negatively charged complexes. Therefore, the formation of one-phase systems should be guaranteed for mixtures of PDADMAC with AKYPO and SLES. For the mixtures of the surfactant with Merquat 2003, a direct electrostatic interaction between the surfactants and the positively charged 3-trimethylammonium propyl methacrylamide chloride monomers was expected, which, considering only the cationic monomers, which should lead again to the formation of negatively charged complexes, with S/P ratio values of about 35 and 25 for the interaction with SLES and AKYPO, respectively. These unexpected pictures can be understood only when considering the formation of polyelectrolyte–surfactant kinetically trapped aggregates. These are the result of the Marangoni stresses created during the mixing process as a result of the local excess of surfactant molecules [49]. The origin of the phase separation for the mixtures of the polymers with LE4 remains unclear, especially taking into account that no electrostatic interaction of LE4 with the polymers was expected, and consequently, the formation of neutral complexes should be prevented within the entire range of surfactant concentrations. However, considering the low solubility of LE4 in water, it may be expected that its interaction with the polymers through non-electrostatic interaction can result in the formation of complexes with a reduced solubity, and hence, a fast precipitation occurred upon the mixture of the polymer with concentrated LE4 solutions.

**Figure 3:**
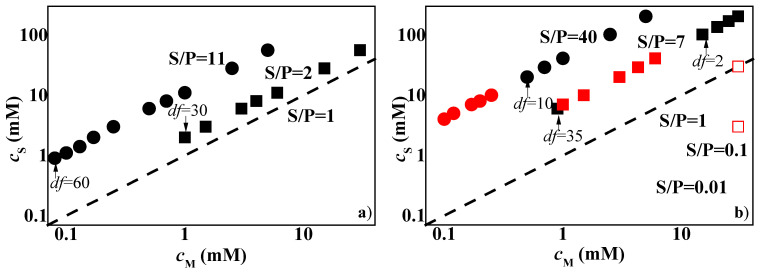
Surfactant (*c*_S_)–polymer (*c*_M_) concentration phase diagrams for the combination of PDADMAC (■ and ■) or Merquat 2003 (● and ●) with LE12 (**a**) and CAPB (Notice that □ corresponds to phase-separated mixtures obtained by mixing a fixed PDADMAC concentration and increasing CAPB concentrations) (**b**). The black and red symbols correspond to one-phase and phase-separated mixtures, respectively, and the discontinuous solid line corresponds to the dilution of a hypothetical mixture with S/P = 1. The values of the dilution factors (*df*) corresponding to the onset and exit of the phase-separation region, or the maximum *df* explored for samples in which only phase was found are also indicated. Furthermore, the S/P values corresponding to the dilution line of each sample are also included. Notice that the data for Merquat 2003 only considers the concentration of cationic monomers.

Once the monophasic and phase-separated character of the pseudo-binary mixtures at the highest concentrations was established, the behavior of the 1ϕ mixtures was studied upon dilution to establish the concentrations at which the systems reached the onset of the phase separation. Figure 3 shows the results for the mixtures of PDADMAC or Merquat 2003 with CAPB and LE12, as a representation of the surfactant concentration, *c*_S_, versus the concentration of monomer units, *c*_M_, (for the case of Merquat 2003, the monomer unit is referring to that of the positively charged monomers). For the sake of comparison in Figure 3, the line corresponding to the dilution of a hypothetical mixture with S/P = 1 is shown, and where it is possible, the values of the dilution factor (*df*) corresponding to the borders of the phase-separation region are also indicated. It should be noted that the dilution factor provides information of the amount of water added to the initial concentrated mixtures. Thus, a dilution factor of 2 indicates that the sample contained the same volumes of the initial concentrated mixture and added water, and a dilution factor of 10 indicates that the volume of the concentrated mixture was reduced by adding water to reach a concentration ten times lower than the initial one.

**Figure 4:**
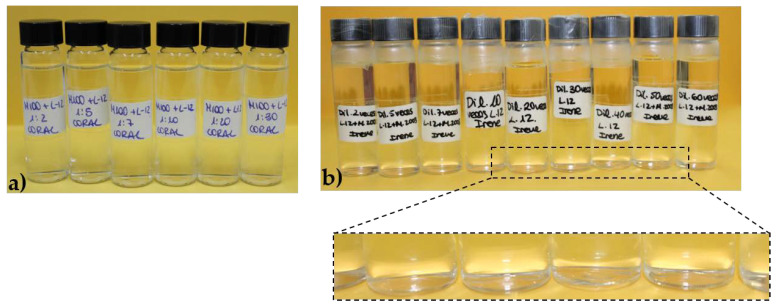
Set of images showing the dilution process (from left to right) of the pseudo-binary polymer–LE12 mixtures with the same composition of the components as that existing in the model mixture: (**a**) PDADMAC–LE12; (**b**) Merquat 2003–LE12. Panel (**b**) shows a more detailed image where the absence of phase separation is clear. In both panels, *df* increases from left to right.

The results for the mixtures of the polymers with LE12 did not show any evidence of phase separation, at least up to a dilution factor (*df*) of 30 and 60 for the PDADMAC and Merquat 2003 mixtures (see Figure 4 in which a set of images showing the aspects of samples of LE12 with PDADMAC and Merquat 2003 with different dilution factors is displayed), respectively. However, considering the neutral character of the surfactant, it could not be clearly stated whether the working concentrations at the phase-separation region had already been exceeded or whether it had not yet been reached, but it was clear that the phase separation, if it occurs, could not be mediated through the neutralization of the polymer charges. Further insights on the complex formation process may be obtained from electrophoretic mobility and surface tension measurements, especially for diluted mixtures [50,51,52]. However, the use of the interpretation of the results obtained from these techniques in concentrated mixtures such as those studied in this work remains challenging.

The situation becomes more interesting for mixtures of PDADMAC or Merquat 2003 with CAPB. The latter is a zwitterionic surfactant that can interact with the positively charged monomers through its anionic terminal group. This association leads to the neutral complexes for surfactant concentrations being close to their critical micellar concentration [33]. According to the above picture, the formation of complexes with an excess of negative charges for the values of the S/P ratio of the samples of PDADMAC (S/P = 7) and Merquat 2003 (S/P = 40) may be expected, which are well above to the hypothetical phase separation line (S/P = 1). However, the results showed that the dilution of the mixtures of CAPB with both polymers drove the system to a precipitation region. This could be explained by the combination of the reduced effective charge of the complexes as consequence of the charge screening associated with the relatively high ionic strength of the complexes, and a non-complete dissociation of the carboxylic head-group of the CAPB molecules. This shifts the charge neutralization line to higher surfactant concentrations, thus leading to phase separation for S/P values far from 1 [14]. The differences in the values of the *df* required for entering into the phase separation for mixtures containing PDADMAC and Merquat 2003 was due to the presence of anionic acrylate monomers in Merquat 2003 that counteract the neutralization process, taking the separation region to higher values of the *df*.

It should be noted that, for the sake of comparison, the phase separation of the mixtures of CAPB with PDADMAC was explored by mixing a fixed polymer concentration (0.5% *w*/*w*) and increasing the surfactant concentrations (in the range 3 × 10^−6^–30 mM). These experiments evidenced that phase separation could also emerge for S/P ratios well below 1 (see Figure 3). This may be ascribed to the formation of kinetically trapped aggregates during the mixing procedures and may present a high significance for designing technical formulations in which the surfactant concentration is minimized.

### 3.2. Study of the Deposition of MonoPhasic Pseudo-Binary Polymer-Surfactant Mixtures

**Figure 5:**
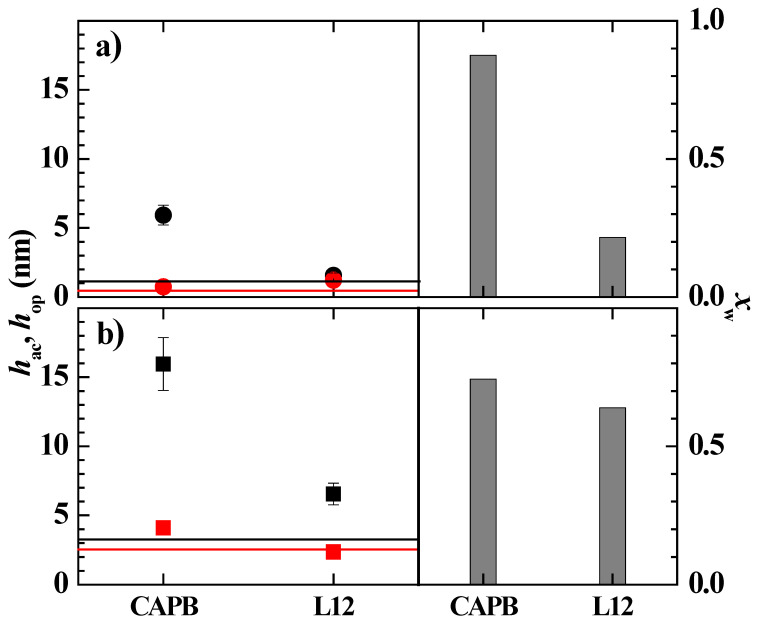
Acoustic (■) and optical thickness (■), and water content (bar charts) for the adsorption of pseudo-binary polymer–surfactant mixtures with the same concentration of the components as in Table 2. (**a**) Mixtures with PDADMAC. (**b**) Mixtures with Merquat 2003. Notice that the solid lines represent the acoustic (―) and optical (―) thicknesses for the adsorption of polymer solutions with the same polymer concentration as that of the polymer–surfactant mixtures.

Figure 5 shows a comparison of the optical, *h*_op_, and acoustic, *h*_ac_, thicknesses, as well as the degree of hydration of the layers obtained by adsorption from solutions of the concentrated pseudo-binary polymer–surfactant mixtures that present a monophasic character when the concentration of the components is the same as in Table 2, i.e., the mixtures of PDADMAC and Merquat 2003 with LE12 and CAPB. 

The results showed that the adsorbed layers of the pseudo-binary mixtures containing Merquat 2003 as the polymer presented a higher adsorption than those corresponding to the mixtures containing PDADMAC. This may be explained considering that the electrostatic repulsions between the carboxylic groups of the Merquat 2003 copolymer and the negatively charged surface force the spreading of the acrylate monomers in a vertical direction to the surface, whereas PDADMAC chains adsorb in a more extended conformation due to the purely cationic nature of this polymer. Furthermore, the adsorption of the mixtures with the neutral surfactant, LE12, was almost negligible in relation to that corresponding to the mixtures containing CAPB. The latter may be explained considering that the association of LE12 with polymers occurred in such a way that the surfactant molecules bound to the polymer chains provoked a strong steric hindrance for the direct interaction of the charged monomers with the negatively charged surface, whereas the association of polymers with CAPB occurred through electrostatic interactions, leading to a situation where each neutralized monomer for the surfactant binding was compensated with a new positive charge corresponding to the surfactant.

Moreover, the complexes obtained through the electrostatic interaction between the carboxylic acid group of the surfactant and the quaternary ammonium groups of the polymers gave rise to more hydrated layers and of higher thicknesses than those corresponding to the pure polymers. Unlike the pseudo-binary systems containing CAPB, where the polymer–surfactant interaction was similar regardless of the polymer used, mixtures containing the neutral surfactant LE12 presented different interactions depending on the specific polymer. Thus, the association of LE12 with PDADMAC occurred mainly through the hydrophobic interaction between the hydrophobic regions of the polymer backbone and the surfactant tails, whereas the association with Merquat 2003 introduced an additional contribution associated with the possibility of hydrogen bonding between the oxyethylene groups of the surfactant tails and the acrylamide and non-dissociated carboxylic acid residues on the polymer backbone. These polymer–surfactant interactions did not modify the interaction of the polymers with the surface, but favored the increase of the hydration degree of the adsorbed layer.

### 3.3. Study of the Deposition Enhanced by Dilution for Pseudo-Binary Polymer–CAPB Mixtures

**Figure 6:**
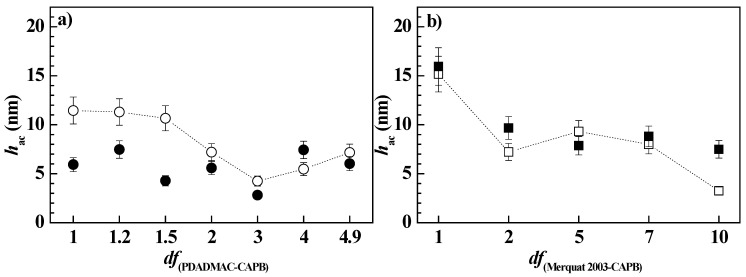
Acoustic thickness before (o and □) and after (● and ■) rinsing/dilution for the adsorption of pseudo-binary polymer–CAPB mixtures obtained upon dilution at different values of *df* of a pseudo-binary polymer–surfactant mixture with the same concentration of the components as those presented in the formulation of Table 2. (**a**) Mixtures with PDADMAC. (**b**) Mixtures with Merquat 2003.

Considering that the dilution of polymer–LE12 mixtures did not take the system toward the phase separation, at least within the explored compositional range, the study of the effect of the dilution of the deposition process of pseudo-binary mixtures was limited to the study of the polymer–CAPB pseudo-binary mixtures. Figure 6 shows the acoustic thicknesses corresponding to the adsorption process and subsequent rinsing of the polymer–CAPB mixtures as a function of the *df* values (Notice that the rinsing process involved a dilution of the initial concentration for a factor close to 5).

The results for the mixtures of the surfactant with both PDADMAC and Merquat 2003 showed a decrease in thickness with dilution, as expected for one-phase diluted mixtures. However, a more detailed analysis evidenced that as the composition was close to *df* values around 3 and 2 for the pseudo-binary mixtures with PDADMAC and Merquat 2003, respectively, there was a slight increase in the adsorption. This may be associated with the onset of the precipitation region as a result of the dilution process. Considering that phase separation occurred about *df*~5 for the PDADMAC–CAPB mixtures and *df*~10 for the Merquat 2003–CAPB one, the results suggested a faster onset of the phase-separation region for the PDADMAC–CAPB mixtures than for mixtures of CAPB with Merquat 2003. In the latter mixtures, a more progressive onset in the phase separation may be expected, with the first signatures of this region appearing for these compositions at around *df* = 2. This difference may be explained considering the larger molecular weight of Merquat 2003 than PDADMAC (the average molecular weight of Merquat 2003 is about six times that of the PDADMAC) which leads to the formation of larger complexes that can sediment more easily than the complexes formed by PDADMAC. However, Merquat 2003–CAPB complexes presents higher colloidal stability than PDADMAC–CAPB ones because of the negative charge associated with the presence of the acrylate monomers in Merquat 2003. This can counteract the charge neutralization due to the electrostatic interaction between the quaternary ammonium groups of the polymer with the carboxylic acid group of the CAPB.

**Figure 7:**
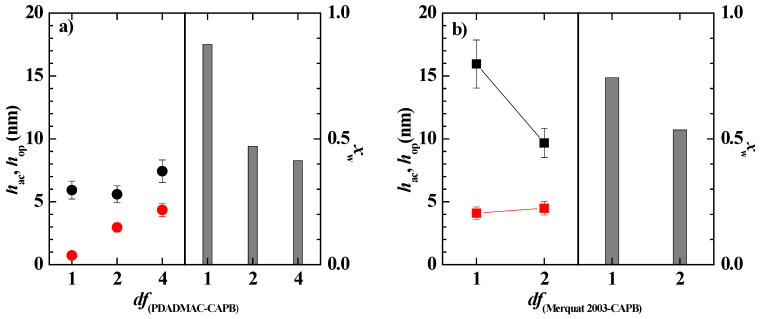
Comparison of the acoustic (● and ■) and optical (● and ■) thickness for the deposition of polymer–CAPB layers, and water content of the deposited layers (bar charts), for samples at different values of the dilution factor. (**a**) Mixtures with PDADMAC. (**b**) Mixtures with Merquat 2003.

Figure 7 displays the comparison between the optical and acoustic thicknesses obtained for the adsorption of polymer–CAPB mixtures, and the water content of the layers, for the representative *df* values. The results confirmed that as the polymer–CAPB mixture draws nearer to the onset of the phase separation, there is an enhanced deposition of solid material as is evidenced from the increase of the optical thickness of the layer. This increase of the layer thickness may be ascribed to the sedimentation of the polymer–CAPB aggregates, which leads to the densification of the layers, making the incorporation of water molecules more difficult, i.e., the water content of the layer decreases (see bar charts in Figure 7a,b).

**Figure 8:**
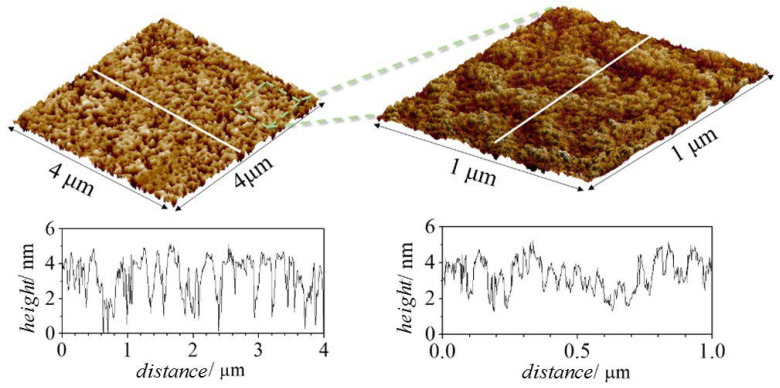
Experimental results obtained by AFM for a layer adsorbed from the concentrated Merquat 2003–CAPB solution of *df* = 1. Three-dimensional topographic images obtained over an area of 4 × 4 μm^2^ using a scanning resolution of 15.6 nm/pt (**left** image), and over an area of 1 × 1 μm^2^ using a scanning resolution of 3.9 nm/pt (**right** image). Below each image is depicted the corresponding height profile performed along the white line.

**Figure 9:**
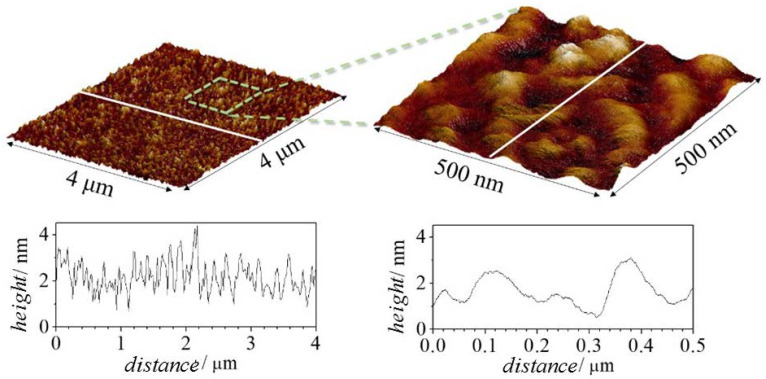
Experimental results obtained by AFM for a layer adsorbed from a diluted Merquat 2003–CAPB solution of *df* = 5. Three-dimensional topographic images obtained over an area of 4 × 4 μm^2^ using a scanning resolution of 15.6 nm/pt (**left** image), and over an area of 0.5 × 0.5 μm^2^ using a scanning resolution of 1.95 nm/pt (**right** image). Below each image is depicted the corresponding height profile performed along the white line.

Atomic force microscopy (AFM) can help in understanding the deposition process of polymer–CAPB layers. Considering the similarities in the behavior of the mixtures containing PDADMAC and Merquat 2003, AFM analysis using tapping mode was focused on in the study of the topography of dried films formed upon deposition of Merquat 2003–CAPB mixtures at two different compositions on a negatively charged silicon dioxide substrate. The first sample corresponded to the film obtained upon the deposition of the pseudo-binary mixture with the same composition of components as the model mixture, i.e., concentrated mixtures (*df =* 1), with the second sample corresponding to the film obtained by the deposition of the concentrated mixture upon dilution by a factor 5 (*df* = 5). This type of study attempted to test whether the morphology of the adsorption layer obtained upon deposition of the concentrated mixture (see Figure 8) differed from the adsorption of diluted mixtures (see Figure 9). As was mentioned above, the dilution process of the mixtures brought the system closer to the phase separation region, with the decrease in surfactant concentration being one of the most critical parameters controlling the onset of the separation region. Therefore, assuming that for *df* = 5 the mixture had a composition close to the onset of the phase separation region, it may be expected that any change in the layer topography associated with dilution should be observed in AFM images.

The AFM images did not show any significant difference in the main characteristics of the layer topographies, with small elevations uniformly distributed along the whole sample appearing independently of the composition of the sample. The roughness of the obtained layers were 0.95 nm and 0.5 nm for the deposition from solutions with df values of 1 and 5, respectively. This decrease of the roughness with the increase of the dilution factor can be understood considering the reduction of the polymer concentration that reduces the layer fuzziness [41]. It should be stressed that the height profiles obtained from the AFM experiments were compatible with the optical thickness values obtained using ellipsometry.

### 3.4. Bulk and Interfacial Behavior of Pseudo-Ternary Mixtures Containing Two Polymers and CAPB

**Figure 10:**
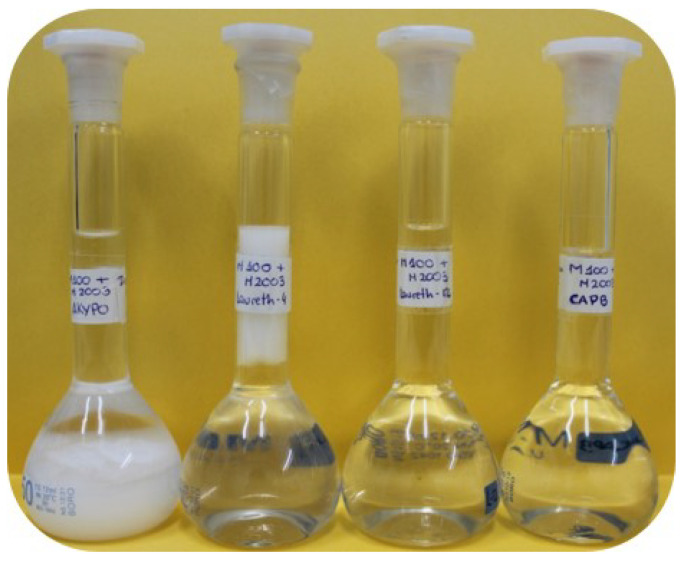
Image showing the aspect of the pseudo-ternary polymer–surfactant mixtures, containing both PDADMAC and Merquat 2003, with the same composition of components as that existing in the model mixture. The mixtures contain the following surfactants (from left to right): AKYPO, LE4, LE12, and CAPB.

It is known that the understanding of the physico-chemical behavior of multicomponent formulations requires one to consider a broad range of variables that lead to the stability of the final product [14]. Thus, according to the results discussed in Section 3.1, it is possible to obtain a one-phase multicomponent mixture despite the pseudo-binary mixtures of two components at the same concentration being a phase-separated system. This section attempts to further our understanding of the properties of the multicomponent mixtures by studying complex pseudo-ternary mixtures. For this purpose, polymer–CAPB mixtures containing both PDADMAC and Merquat 2003 at the same concentrations as those in Table 2, i.e., 0.5% *w*/*w* and 0.25% *w*/*w* for PDADMAC and Merquat 2003, respectively, were created. Figure 10 shows pictures of the samples of these mixtures containing PDADMAC, Merquat 2003, and different surfactants.

The presence of the two polymers made it possible to obtain monophasic mixtures for mixtures containing LE12, which was not possible for pseudo-binary systems. This suggested the existence of specific synergistic interactions between the two polymers which favor the solubilization of the surfactant, even though the addition of the LE12 to solutions of individual polymers resulted in phase separation. Therefore, it is possible that the presence of specific synergistic interactions between the components of the mixtures are the mechanism underlying the preparation of one-phase technical formulations.

**Figure 11:**
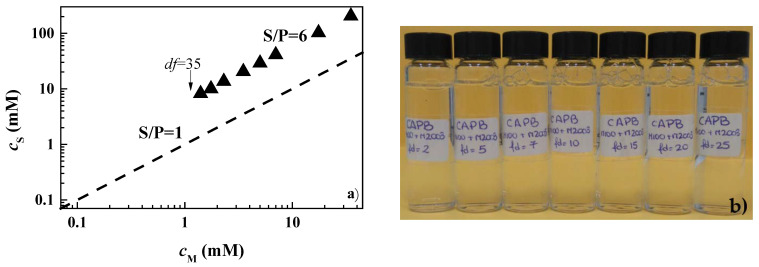
(**a**) Surfactant–polymer concentration phase diagrams for the combination of PDADMAC, Merquat 2003, and CAPB. The symbols correspond to one-phase mixtures and the discontinuous solid line corresponds to the dilution of a hypothetical mixture with S/P = 1. The value of the dilution factors (*df*) corresponding to the maximum *df* explored is also indicated. Furthermore, the S/P value corresponding to the dilution line of the sample is also included. Notice that polymer concentration is referring to the total concentration of cationic monomers, including those corresponding to both polymers. (**b**) Image showing the dilution process (from left to right) of the pseudo-ternary polymer–CAPB mixtures with the same composition of components as in Table 2.

Considering that the adsorption of pseudo-binary mixtures with LE4 was not significant, it was decided to focus the study on the behavior of the pseudo-ternary mixtures containing CAPB as the surfactant. First, we studied the dilution diagram in terms of a representation similar to that used in Figure 3 for pseudo-binary mixtures (see Figure 11a).

The results showed that the simultaneous mixture of CAPB with the two polymers led to a possible shift of the phase-separation region in relation to that found in the pseudo-binary mixtures. This was clear when considering that the dilution factor assumes a value of 2 and 10 for the onset of the phase separation of mixtures of CAPB with PDAMAC and Merquat 2003, respectively, whereas for the pseudo-ternary mixture no evidence of phase separation was found even for *df* values of about 35. This was a surprising result, especially considering that the S/P ratio was closer to the phase separation line (S/P = 1) than that corresponding to the pseudo-binary mixtures, and suggested that the simultaneous interaction of both polymers with the surfactants enhanced the solubility of the polymer–surfactant aggregates. Figure 11b shows a set of pictures including solutions with increasing *df* values for the mixture of the two polymers and CAPB.

**Figure 12:**
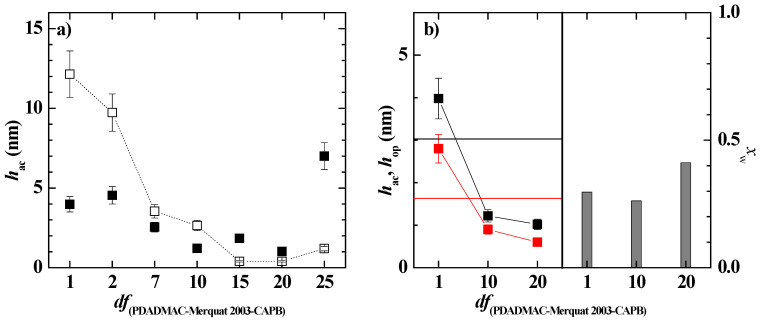
(**a**) Acoustic thickness before (□) and after (■) rinsing/dilution for the adsorption of pseudo-ternary polymer–CAPB mixtures obtained upon dilution at different values of *df* of a pseudo-ternary polymer–surfactant mixtures with the same concentrations of the individual components as those that are presented in the formulation of Table 2 (**b**) Comparison of the acoustic (■) and optical (■) thicknesses for the deposition of layers of the polymer–CAPB pseudo-ternary mixtures, and water content of the deposited layers (bar charts), for samples at different values of the dilution factor. Notice that the solid lines represent the acoustic (―) and optical (―) thicknesses for the adsorption of a solution containing a mixture of the two polymers in the same concentration as in Table 2, i.e., a concentration of polymer equivalent to *df* = 1.

Figure 12 shows the dependence of the acoustic thicknesses, before and after washing, on the dilution factor for the pseudo-ternary polymer–surfactant mixture, showing that the mixing of both polymers with CAPB shifted the approach to the phase region to higher dilution values (*df*~15) than those corresponding to the pseudo-binary mixtures. This is in agreement with the absence of phase separation within the explored dilution range (see Figure 11), and can be ascribed to the existence of synergistic interaction between the two polymers that favors the solubilization of the aggregates; thus, enhancing their colloidal stability and hence, delaying their precipitation [26,53].

The results showed that the adsorbed layer of the concentrated pseudo-ternary mixture (*df* = 1) appeared thicker than the layer corresponding to the bare polymer mixture, i.e., the Merquat 2003–PDADMAC mixture. This is due to the multiple electrostatic interactions occurring between the molecules in the solution, including those between polymer and CAPB, and PDADMAC and Merquat 2003, that leads to a reduction of both the number of charged groups interacting with the surface and the repulsion between the polymer chains. The latter favors the adsorption of compacted aggregates, thus optimizing the interaction between the charged monomers and the surface. It should be noted that a comparison of the results in Figure 7 and Figure 12 suggested that the main contribution to the layer thickness of the mixture is the Merquat 2003. However, the results also evidenced that the mixture of both polymers presented an antagonistic effect with respect to the adsorption of the pseudo-binary mixture Merquat 2003–CAPB.

### 3.5. Behavior of the Model Mixture upon Dilution: Towards the Real Application

**Figure 13:**
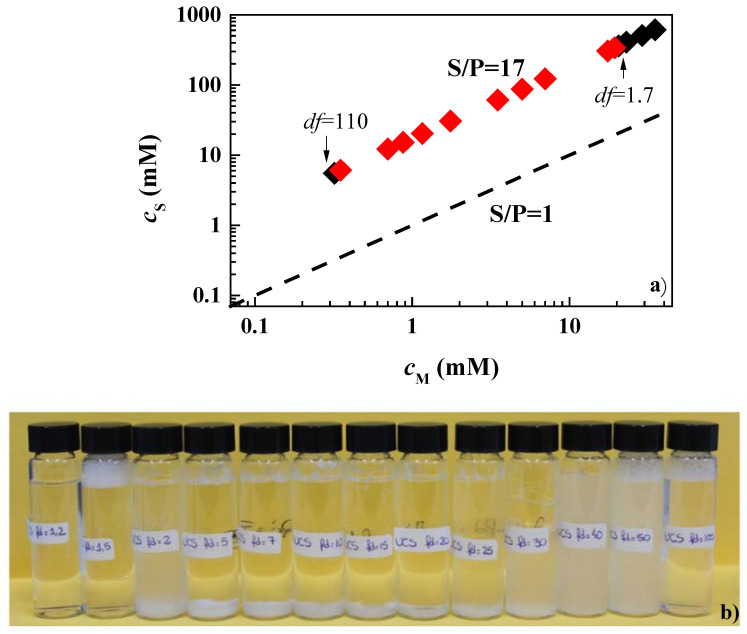
(**a**) Surfactant–polymer concentration phase diagrams for the multicomponent mixture studied. The black and red symbols correspond to one-phase and phase-separate mixtures, respectively, and the discontinuous solid line corresponds to the dilution of a hypothetical mixture with S/P = 1. The value of the dilution factors (*df*) corresponding to the onset and exit of the phase separation region is also indicated. Furthermore, the S/P value corresponding to the dilution line of the sample is also included. Notice that polymer concentration is referring to the total concentration of cationic monomers, including those corresponding to both polymers. (**b**) Image showing the dilution process (from left to right) of the model mixture. The total composition is given in Table 2.

In the previous sections, the phase behavior and the adsorption of pseudo-binary and pseudo-ternary polymer–surfactant mixtures with concentrations of the components similar to those found in the model mixture was discussed. In what follows, the behavior of a multicomponent mixture upon dilution will be explored. This will provide information about the potential effectiveness of a commercial shampoo under real application conditions. The composition of the mixture is given in Table 2. The results showed that the model mixture was highly optimized, undergoing phase separation upon dilution at relatively low values of the dilution factor (see Figure 13). Furthermore, the phase-separation region was extended across *df* values spanning almost two orders of magnitude greater.

**Figure 14:**
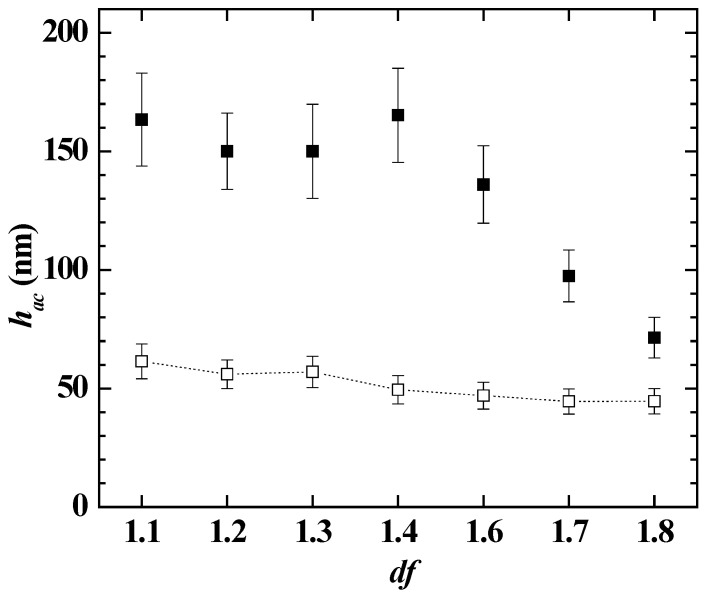
Acoustic thickness before (□) and after (■) rinsing/dilution for the adsorption of model mixture at different values of *df*.

The above results were in agreement with the picture found upon the adsorption and subsequent rinsing of solutions with different values of *df* as shown Figure 14. Thus, the rinsing of the adsorption layer corresponding to the concentrated model mixture (corresponding to *df* = 1) drove it to an enhanced deposition, i.e., a strong thickness increase, leading to a layer with a larger thickness than those corresponding to the explored pseudo-binary and pseudo-ternary mixtures. This may be the result of the multiple interactions occurring between the different components contained in the model mixture and their high concentration. The increase in the adsorption upon rinsing decreased as the dilution factor increased, which can be ascribed to the formation of denser layers.

**Figure 15:**
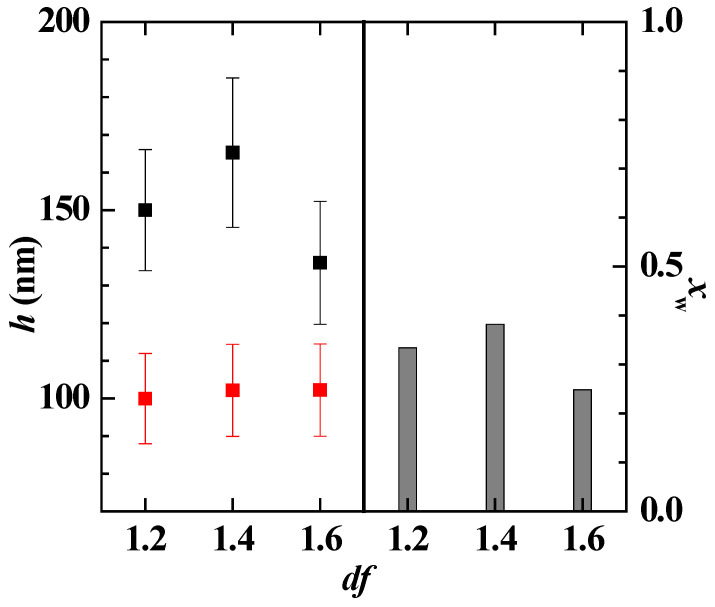
Comparison of the acoustic (■) and optical (■) thicknesses for the deposition of layers of the experimental model mixture, and water content of the deposited layers (bar charts), for samples at different values of the dilution factor.

Figure 15 shows the comparison of the acoustic and optical thicknesses obtained and the degree of hydration the layers obtained upon the adsorption and subsequent rinsing of the model mixture at different *df* values. These results confirmed the abovementioned densification of the layer occurring as the *df* increases, i.e., the reduction of the water content.

It must be noted that the dilution of the complex formulation leads to very thick adsorbed layers, over 100 nm, which, depending of the polymers and surfactants used, can strongly improve the smoothness of the hair fibers after dilution of the formula under the shower. This is clearly an indication of the importance of knowing the phase diagram of this type of mixture. Unfortunately, its theoretical prediction is still not possible.

## 4. Conclusions

We attempted to shed light on some of the most fundamental aspects governing the performance of multicomponent polyelectrolyte–surfactant mixtures under conditions relevant for their application. For this purpose, we explored the effect of the dilution in the phase behavior and deposition onto negatively charged surfaces, similar to those found in the external region of the cuticle or in rocky reservoirs during oil recovery, of pseudo-binary and pseudo-ternary polymer–surfactant mixtures and a multicomponent polymer–surfactant mixture. This is very relevant for the rational design and optimization of formulations for different technological applications.

The results showed that multicomponent technical mixtures present an optimized composition, resulting in an enhanced deposition upon a slight dilution, which is due to the fact that its composition is close to that corresponding to the phase-separation region. This is worth knowing for ensuring the effectiveness of the products by facilitating the precipitation of the polyelectrolyte–surfactant complex without needing to use large quantities of water, which favors their action mechanism. On the other hand, the study of pseudo-binary and pseudo-ternary mixtures with concentrations of the components close to those used in multicomponent technical formulations evidenced that simpler mixtures did not provide a true representation of the final solubility profiles of muticomponent polyelectrolyte–surfactant mixtures, with their phase behavior and response upon dilution emerging far from what was found for the multicomponent mixtures. This suggested that in multicomponent systems, such as shampoo, there exists complex synergistic interactions between the components, favoring their solubility, and hence, the colloidal stability of the mixtures. Therefore, the results of this study suggested that the behavior of shampoos, and their conditioning properties, cannot be solely explained in terms of the behavior of simpler mixtures including only two or three of the shampoo components. However, this type of study helps in the understanding of the predeposition conditions, enabling a choice of the most suitable combination of polymers and surfactants for the design of formulations for specific applications.

## Figures and Tables

**Table 1:** Polymer and surfactant contained in a model shampoo according to reference [29].

Component	Type of Molecule
copolymer of acrylamide and diallyldimethylammonium chloride	Polymers
copolymer of styrene and acrylates	
tocopheryl acetate	Surfactants
lauryl ether (4 oxyethylene groups)	
sodium lauryl ether sulfate	
Cocoisopropanolamide	
cocoamidopropyl betaine	
polyethylenglycolether of glyceryl cocoate (7 oxyethylene groups)	

**Table 2:** Composition of the technical model’s formulation designed in this study.

Component	Concentration	Type of Molecule
PDADMAC	0.5% *w*/*w* ^1^	Polymers
Merquat 2003	0.25% *w*/*w* ^2^	
lauryl ether (12 oxyethylene groups)	56 mM	Surfactants
lauryl ether (4 oxyethylene groups)	28 mM	
sodium lauryl ether sulfate	186 mM	
cocamidopropyl Betaine	204 mM	
laureth-5 carboxylic acid	136 mM	

^1^ The concentration of PDADMAC referring to monomer units is 30 mM. ^2^ The concentration of Merquat 2003 referring to positively charged monomer units is 5 mM.

## Data Availability

Data are available upon request.
